# Tunable control of CAR T cell activity through tetracycline mediated disruption of protein–protein interaction

**DOI:** 10.1038/s41598-021-01418-9

**Published:** 2021-11-09

**Authors:** Alastair Hotblack, Evangelia K. Kokalaki, Morgan J. Palton, Gordon Weng-Kit Cheung, Iwan P. Williams, Somayya Manzoor, Thomas I. Grothier, Alice Piapi, Valeria Fiaccadori, Patrycja Wawrzyniecka, Harriet A. Roddy, Giulia Agliardi, Claire Roddie, Shimobi Onuoha, Simon Thomas, Shaun Cordoba, Martin Pule

**Affiliations:** 1 Department of Haematology, UCL Cancer Institute, University College, 72 Huntley Street, London, WC1E 6DD UK; 2 Autolus Therapeutics, White City, London, UK

**Keywords:** Cancer immunotherapy, Haematological cancer, Immunotherapy, Molecular engineering

## Abstract

Chimeric antigen receptor (CAR) T cells are a promising form of cancer immunotherapy, although they are often associated with severe toxicities. Here, we present a split-CAR design incorporating separate antigen recognition and intracellular signaling domains. These exploit the binding between the tetracycline repressor protein and a small peptide sequence (TIP) to spontaneously assemble as a functional CAR. Addition of the FDA-approved, small molecule antibiotic minocycline, acts as an “off-switch” by displacing the signaling domain and down-tuning CAR T activity. Here we describe the optimization of this split-CAR approach to generate a CAR in which cytotoxicity, cytokine secretion and proliferation can be inhibited in a dose-dependent and reversible manner. Inhibition is effective during on-going CAR T cell activation and inhibits activation and tumor control in vivo*.* This work shows how optimization of split-CAR structure affects function and adds a novel design allowing easy CAR inhibition through an FDA-approved small molecule.

## Introduction

Chimeric antigen receptor (CAR) therapies are an effective treatment for relapsed/refractory B cell malignancies^1,2^ and myeloma^3^ and are under development in solid cancers^4,5^. However, use of CAR T cell therapies is also associated with significant toxicity such as cytokine release syndrome (CRS) and immune effector cell associated neurotoxicity syndrome (ICANS)^6^. Whilst tocilizumab or high-dose corticosteroids are effective at controlling CRS^6,7^, they are less effective at treating ICANS^8,9^. Moreover, as CAR T cell therapy is expanded to other settings, including solid tumors, the new targets for these are often tumor-associated, rather than tumor-specific and so increase the risk of on-target, off-tumor toxicities.

In contrast to small molecule or protein therapeutics, CAR T cells engraft and expand so that toxicity can be progressive and fulminant. Consequently, genetically encodable safety mechanisms, which can control CAR T cell activity after administration of a pharmaceutical control agent, have been developed. For instance, several suicide genes have been described which allow selective destruction of CAR T cells in the face of toxicity^10–12^. Whilst this strategy allows for control, CAR T cells are permanently lost, which, depending on the timing and clinical setting, could cause disease relapse^13^.

Subsequent approaches have aimed to develop CARs which can offer titratable and reversible control of CAR T activity. In this family are split-CAR “on” designs, where the presence of a dimerizing molecule is needed to link the target binding domain to a signaling domain^14–16^. In this setting, withdrawal of the dimerizing pharmaceutical leads to CAR deactivation. A limitation of this approach is the dependence on variable pharmacokinetics and bio-distribution of a chronically administered pharmaceutical as well as inconvenience associated with the requirement for continual dosing of these drugs.

A more convenient approach is a split CAR “off” design whereby a small molecule pharmaceutical disrupts the interaction between the target binding and signaling domains. With this approach, CAR is deactivated following administration of the small molecule. Such an approach has recently been described by Giordano-Attianese et al.^17^. Here, a synthetic heterodimeric CAR was designed which could be disrupted by addition of a small molecule drug. However, a limitation of this system was that a novel, clinically untested small molecule was required, limiting the ease of clinical translation of such an approach.

Here, we sought to develop a split-CAR “off” system in which the CAR could be dissociated by the addition of a widely available small molecule—minocycline. To this end, we exploited the interaction between the tetracycline repressor protein (TetRB) and the tetracycline peptide mimic TIP to create a split CAR which could be switched off after exposure to minocycline (TetCAR). Iterative optimization identified a TetCAR format which incorporated a Fab binding domain and CD28-endodomain. This format maintained potent activity in the absence of minocycline with reversible, minocycline-induced inhibition.

## Results

### A TIP CAR efficiently associates within cells and dissociates upon addition of small molecule

**Figure 1:**
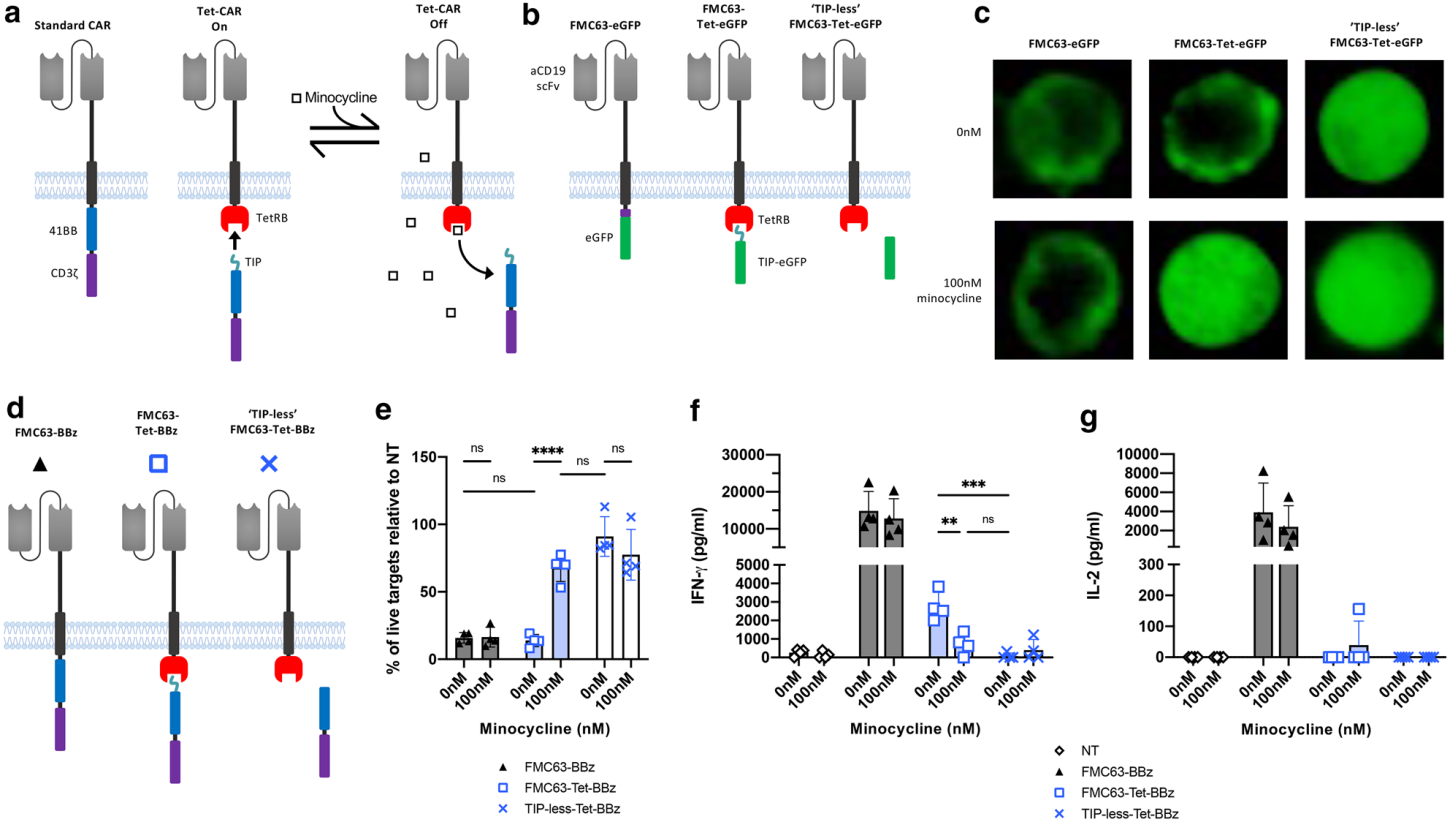
A split CAR associates through the interaction of TetRB and TIP and dissociates upon minocycline addition. (**a**) Overview of the split CAR approach (TetCAR), incorporating the tetracycline repressor protein B (TetRB) and the peptide TIP. Addition of the small molecule antibiotic minocycline reversibly disrupts TetRB-TIP binding, displaces the endodomain and inhibits CAR activation. (**b**) Schematic of the CAR constructs with eGFP endodomains. CARs contain an anti-human CD19 scFv from FMC63, CD8 stalk regions, CD28 transmembrane domains and eGFP endodomain. TetCARs have a TetRB endodomain with eGFP as a separate protein with or without TIP. (**c**) Representative widefield fluorescent images of HEK293T cells transduced with eGFP-tagged CAR structures, ± 100 nM minocycline. (**d**) Schematic of the CAR constructs with 41BB-CD3ζ endodomains. CARs contain an anti-human CD19 scFv from FMC63, with a CD8 stalk and transmembrane domain and 41BB-CD3ζ endodomain. TetCARs have a TetRB endodomain with 41BB-CD3ζ as a separate protein, with or without TIP. E) Killing of SupT1 cells engineered to express CD19 and GFP (SupT1-CD19-GFP) after 24 h co-culture with CAR-T cells at a 1:1 effector:target ratio. 100 nM of minocycline was added to relevant wells. Data shows mean percentage (± SD) of live cells compared to non-transduced (NT) T-cell control, n = 4 donors from 1 experiment. Statistical analysis was through a two-way ANOVA with Tukey’s multiple comparisons between each group at 0 nM, or with Šidák’s multiple comparisons within each group ± minocycline. *P* values = FMC63-Tet-BBz 0 nM versus 100 nM (****, < 0.0001). (**f**) IFN-γ or (**g**) IL-2 release after 24 h of co-culture with SupT1-CD19-GFP at 1:1 E:T ratio. Data shows mean ± SD, n = 4 donors from 1 experiment. Statistical analysis was through a two-way ANOVA with Tukey’s multiple comparisons between FMC63-Tet-BBz and TIP-less-Tet-BBz. *P* values = FMC63-Tet-BBz 0 nM versus 100 nM (**, 0.0013) and FMC63-Tet-BBz 0 nM versus TIP-less-Tet-BBz 0 nM (***, 0.0001).

TIP is a 16 amino acid peptide mimic of tetracycline which binds to the tetracycline repressor protein B (TetRB) with lower affinity than tetracycline^18,19^. We hypothesized that a split CAR incorporating TIP/TetRB could allow tetracycline-inducible dissociation of the signaling component from the antigen recognition component [Fig. 1a]. As an initial exploration, we generated an anti-CD19 CAR ectodomain incorporating the FMC63 single chain variable fragment (scFv)^20^ with a TetRB endodomain, co-expressed with a separate cytoplasmic TIP-eGFP fusion [Fig. 1b, and Supplementary Fig. S1(a)]. As controls, a similar split construct was generated lacking TIP as well as a “monolithic” CAR with an eGFP endodomain. Microscopy revealed that eGFP localized to the cell membrane in both the monolithic CAR and the split CAR, but not in the split CAR which lacked TIP. In the presence of 100 nM minocycline, the TIP-eGFP was displaced from the membrane and eGFP signal was dispersed throughout the cytoplasm [Fig. 1c]. These data suggested that the TIP/TetRB system could be used for inducible translocation of signaling domains from the cell membrane.

### TetRB/TIP CAR can be controlled by minocycline but has impaired maximal function

To functionally test the split TetRB CAR (‘TetCAR’) approach, the eGFP domains were replaced with 41BB-CD3ζ [Fig. 1d and Supplementary Fig. S1(b)]. Transduced PBMCs were co-cultured for 24 h with SupT1 cells expressing CD19-eGFP in the presence or absence of minocycline. Both standard “monolithic” CAR and the 41BB-CD3ζ TetCAR T cells efficiently lysed SupT1-CD19-GFP targets [Fig. 1e and Supplementary Fig. S1(c) and (d)]. However, whilst addition of minocycline had no effect on the control CAR, cytotoxicity was impaired after addition of minocycline to the TetCAR. There was no significant difference in cytotoxicity between the inhibited TetCAR and the TetCAR control that lacked the TIP sequence, suggesting 100 nM minocycline was sufficient to fully dissociate the TetCAR signaling domains. Secretion of IFN-γ and IL-2 was also quantified after co-culture [Fig. 1f,g]. In line with the cytotoxicity data, addition of minocycline had no effect on cytokine secretion by the control CAR, but significantly reduced IFN-γ secretion by the TetCAR. In the absence of Minocycline however, TetCAR secreted considerably less IFN-γ than the control monolithic CAR and IL-2 secretion was undetectable. These data show that addition of minocycline could inhibit the activation of the TetCAR; however, activity of the uninhibited TetCAR was less than that of a monolithic CAR.

### Fab format and TetRB-attached CD28 co-stimulation improve TetCAR activity

**Figure 2:**
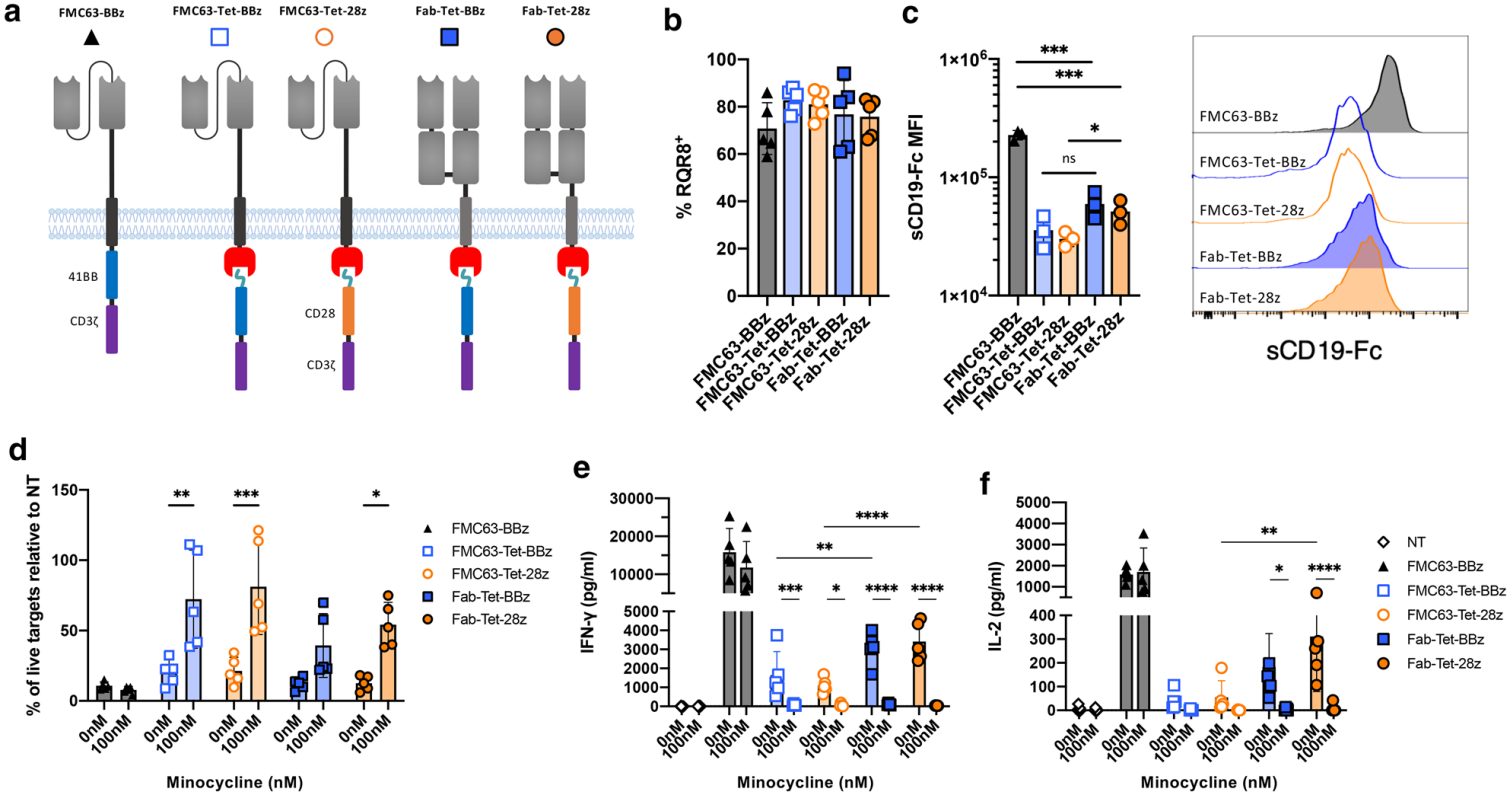
Optimization of TetCAR surface expression and signaling. (**a**) Schematic overview of TetCAR constructs containing 41BB-ζ or CD28-ζ endodomains. Antigen recognition is provided by the FMC63 scFv or Fab fragment. (**b**) Transduction efficiency as measured by CD34 staining of the RQR8 marker gene. Data shows mean ± SD, n = 5 donors from 2 independent experiments. (**c**) Median fluorescent intensity (left) and representative histograms (right) of CAR expression on surface of RQR8^+^ cells as measured by staining with soluble, Fc-tagged CD19 protein. Data shows mean ± SD, n = 3 donors from 1 experiment. Unpaired T tests were used for statistical analysis. *P* values = FMC63-BBz versus Fab-Tet-BBz (***, 0.0003) or Fab-Tet-28z (***, 0.0002), FMC63-Tet-BBz versus Fab-Tet-BBz (ns, 0.089), FMC63-Tet-28z versus Fab-Tet-28z (*, 0.045). (**d**) Killing of SupT1-CD19-GFP after 24 h co-culture with CAR-T cells at a 1:1 effector:target ratio. 100 nM of minocycline was added to relevant wells. Data shows mean percentage (± SD) of live cells compared to non-transduced (NT) control, n = 5 donors from 2 independent experiments. Statistical analysis was through a two-way ANOVA with Šidák’s multiple comparisons within each group ± minocycline. *P* values for each construct + /- minocycline were: FMC63-Tet-BBz (**, 0.0023), FMC63-Tet-28z (***, 0.0003) and Fab-Tet-28z (*, 0.0279). (**e**) IFN-γ and (**f**) IL-2 release after 24 h of co-culture with SupT1-CD19-GFP at 1:1 E:T ratio. Data shows mean ± SD, n = 5 donors from 2 independent experiments. Statistical analysis was through two-way ANOVAs between the TetCAR groups at 0 nM minocycline (with Tukey’s multiple comparisons) or within these groups ± minocycline (with Šidák’s multiple comparisons). *P* values were; between FMC63-Tet-BBz and Fab-Tet-BBz (**, 0.0097, IFN-γ) and between FMC63-Tet-28z and Fab-Tet-28z (****, < 0.0001, IFN-γ and **, 0.0017, IL-2). *P* values between the TetCAR constructs ± minocycline were: FMC63-Tet-BBz (***, 0.0007, IFN-γ), FMC63-Tet-28z (*, 0.0409, IFN-γ), Fab-Tet-BBz (****, < 0.0001, IFN-γ and *, 0.0152, IL-2) and Fab-Tet-28z (****, < 0.0001 both IFN-γ and IL-2).

To enhance maximal performance of TetCAR, several structural modifications were explored. We first tested incorporatation of the CD28 endodomain alongside different transmembrane domains or different linkers between the transmembrane domain and TetRB [Supplementary Fig. S2(a)]. These modifications had no improvement on TetCAR function [Supplementary Fig. S2(b)]. From the previous experiment, we noted that surface expression of TetCAR, as determined by recombinant CD19 binding, was lower than that of monolithic CAR [Supplementary Fig. S2(c) and (d)]. To increase the stability of TetCAR, further variants were constructed in a Fab-CAR format^21^ (Fab-TetCAR, outlined in Fig. 2a and Supplementary Fig. S2(e)), with either 41BB-CD3ζ or CD28-CD3ζ endodomains. Surface expression was increased in both Fab-TetCARs [Fig. 2b,c]. No differences in cytotoxicity of any variant were noted [Fig. 2d]. Inhibition of cytotoxicity was observed upon addition of minocycline in all variants, but this was only significant in the Fab-Tet-28z. Whilst Fab-TetCAR induced IFN-γ and IL-2 secretion were still lower in both variants compared with the control monolithic CAR, the Fab-TetCAR variants secreted higher levels of IFN-γ and IL-2 than their scFv-counterparts [Fig. 2e,f]. Despite the higher baseline levels of cytokine secretion in the Fab-TetCARs, addition of minocycline was still able to potently suppress cytokine secretion in both endodomain variants.

### Addition of a membrane-proximal non-dissociating co-stimulatory domain further enhances cytokine secretion

**Figure 3:**
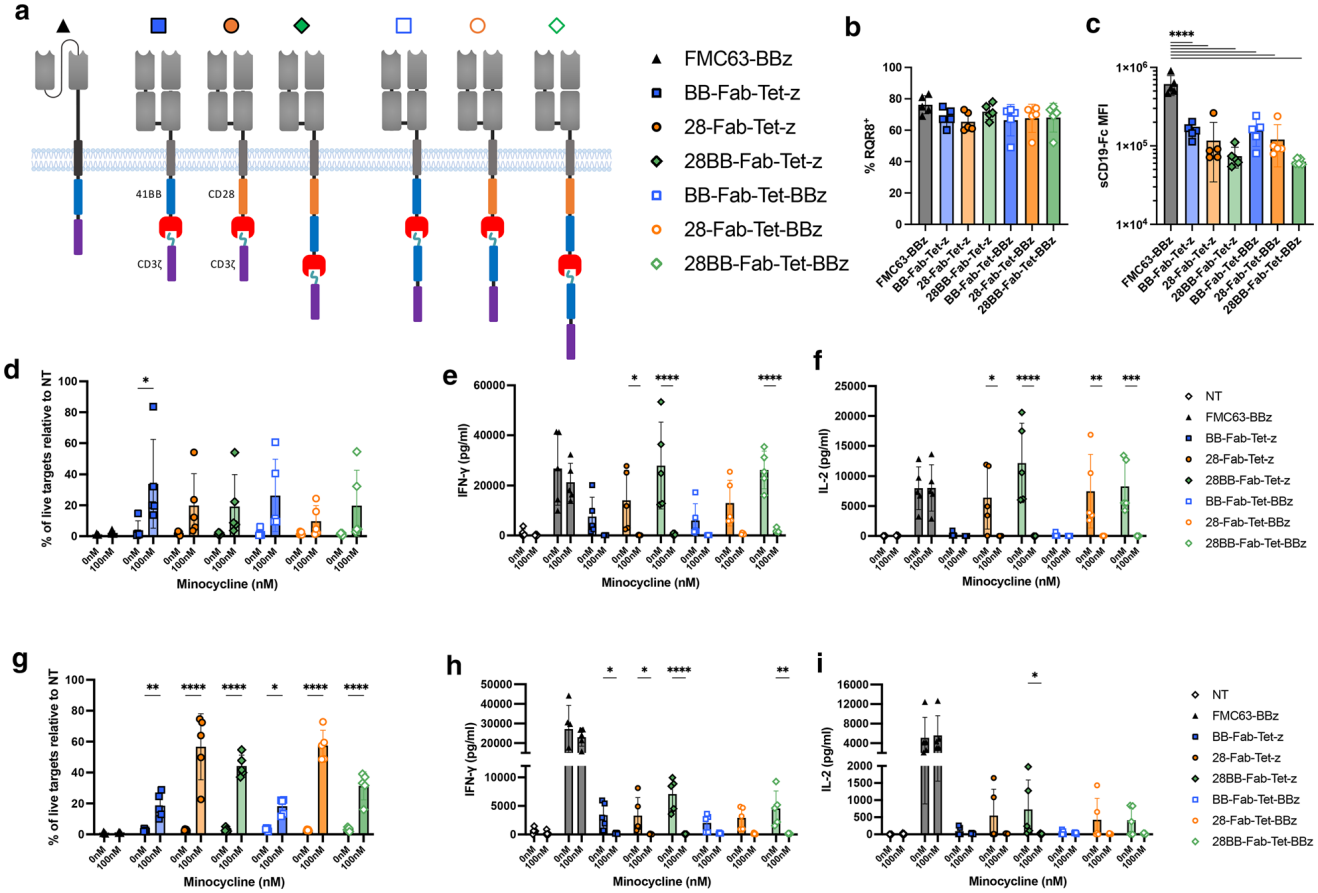
Reconfiguration of endodomain positions enhances TetCAR function. (**a**) Schematic overview of Fab-TetCAR constructs containing membrane-proximal 41BB or CD28 endodomains, with a TIP-CD3ζ or TIP-41BB-CD3ζ domains. (**b**) Transduction efficiency as measured by CD34 staining of the RQR8 marker gene. Data shows mean ± SD, n = 5 donors from 2 independent experiments. (**c**) Median fluorescent intensity of CAR expression on surface of RQR8^+^ cells as measured by staining with soluble, Fc-tagged CD19 protein. Data shows mean ± SD, n = 5 donors from 2 experiments. Statistical analysis was through one-way ANOVA between the groups, *p* values were; between FMC63-BBz and each Fab-TetCAR (****, < 0.0001). Differences between Fab-TetCARs alone were analyzed by one-way ANOVA but were not significant. (**d**) Killing of SupT1-CD19-GFP after 24 h co-culture with CAR-T cells at 1:1 E:T ratio. 100 nM of minocycline was added to relevant wells. Data shows mean ± SD, n = 5 donors from 2 independent experiments. Statistical analysis was through a two-way ANOVA comparing each group ± minocycline (with Šidák’s multiple comparisons). *P* values were; FMC-Tet-BBz (*, 0.0119). (**e**) IFN-γ and (**f**) IL-2 release after 24 h of co-culture with SupT1-CD19 at 1:1 E:T ratio (± 100 nM minocycline). Data shows mean ± SD, n = 5 donors from 2 independent experiments. Statistical analysis was through a 2-way ANOVA comparing each group ± minocycline (with Šidák’s multiple comparisons). *P* values for IFN-γ = 28-Tet-z (*, 0.0484), 28BB-Fab-Tet-z and 28BB-Fab-Tet-BBz (****, < 0.0001). *P* values for IL-2 = 28-Tet-z (*, 0.0150), 28BB-Fab-Tet-z (****, < 0.0001), 28-Fab-Tet-BBz (**, 0.0029) and 28BB-Fab-Tet-BBz (***, 0.0007). (**g**) Killing of NALM6 after 48 h co-culture with CAR-T cells at 1:1 E:T ratio. 100 nM of minocycline was added to relevant wells. Data shows mean ± SD, n = 5 donors from 2 independent experiments. Statistical analysis was through a two-way ANOVA comparing each group ± minocycline (with Šidák’s multiple comparisons). *P* values were; BB-Fab-Tet-z (**, 0.0083), BB-Fab-Tet-BBz (*, 0.0189) and 28-Fab-Tet-z, 28BB-Fab-Tet-z, 28-Fab-Tet-BBz and 28BB-Fab-Tet-BBz (****, < 0.0001). (**h**) IFN-γ and I) IL-2 release after 48 h of co-culture with NALM6 at 1:1 E:T ratio (± 100 nM minocycline). Data shows mean ± SD, n = 5 donors from 2 independent experiments. Statistical analysis was through a 2-way ANOVA comparing each group ± minocycline (with Šidák’s multiple comparisons). *P* values for IFN-γ = BB-Fab-Tet-z (*, 0.0433), 28-Fab-Tet-z (*, 0.0387), 28BB-Fab-Tet-z (****, < 0.0001) and 28BB-Fab-Tet-BBz (**, 0.0020). *P* values for IL-2 = 28BB-Fab-Tet-z (*, 0.0450).

Whilst maximal TetCAR cytokine release was improved by the Fab format, a deficit still remained. We reasoned that a TIP-tethered co-stimulatory domain signals ineffectively and we next introduced co-stimulatory domains between the transmembrane domain and TetRB, with and without additional TIP-tethered co-stimulation (outlined in Fig. 3a). As before, despite similar transduction efficiencies, CAR surface expression was significantly reduced in the Fab-TetCAR variants compared to the monolithic CAR, although differences between the Fab-TetCARs were not significant [Fig. 3b,c]. These variants were tested as before. All TetCAR variants induced similar cytotoxicity, although minocycline induced inhibition of cytotoxicity was less pronounced and was only significant with BB-Fab-Tet-z [Fig. 3d]. However, in contrast to 41BB, introduction of membrane proximal CD28 co-stimulatory domains restored cytokine secretion to that of the control monolithic CAR [Fig. 3e,f]. Notably, minocycline completely inhibited IFN-γ and IL-2 secretion from all Fab-TetCAR T cells, including the variant with CD28 and 41BB endodomains (28BB-Fab-Tet-z). This experiment was also performed using NALM6 cells as targets. The findings were similar to SupT1-CD19, although here cytotoxicity was significantly inhibited in all Fab-TetCARs after addition of minocycline [Fig. 3g]. However, cytokine release in response to NALM6, even by 28BB-Fab-Tet-z CAR, was reduced compared to the monolithic CAR [Fig. 3h,i], likely due to lower CD19 expression in NALM6 cells [Supplementary Fig. S2f].

### Tunable control of optimized TetCAR activity through dose-dependent, minocycline inhibition

**Figure 4:**
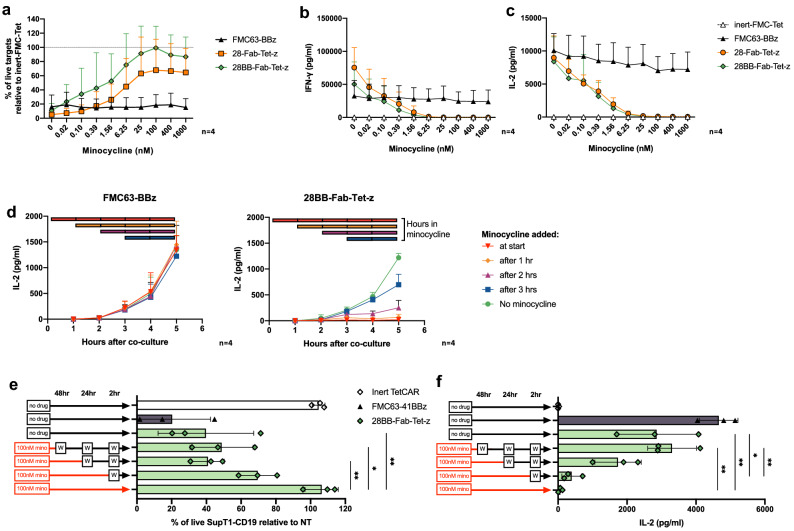
TetCAR activity can be fine-tuned in vitro with a dose-dependent response to minocycline. (**a**) Killing of SupT1-CD19-GFP after 24 h of co-culture with CAR-T cells at 1:4 E:T ratio. A range of minocycline doses from 0.02-1600 nM were added to relevant wells. Data shows mean % of live targets relative to an inert TetCAR control, ± SD. n = 4 donors from 1 experiment. (**b**) IFN-γ and (**c**) IL-2 release after 24 h of co-culture with SupT1-CD19-GFP at various minocycline doses. Data shows mean ± SD, n = 4 donors from 1 experiment. (**d**) IL-2 secretion from FMC63-BBz or 28BB-Fab-Tet-z CARs 1–5 h after co-culture with SupT1-CD19-GFP at a 2:1 E:T ratio. 100 nM minocycline was added to separate wells every hour. Data shows the mean (± SD) secretion of IL-2 at each time-point in groups that received minocycline at the beginning of the experiment, or every hour afterwards. Color coded bars indicate the number of hours that the co-cultures were exposed to minocycline for. n = 4 donors from 2 independent experiments. (**e**) Cytotoxicity or (**f**) IL-2 secretion by 28BB-Fab-Tet-z CARs after coculture with SupT1-CD19 at a 1:1 E:T ratio. Inhibition by minocycline was removed by washing cells with complete media at 48, 24 and 2 h before addition of SupT1-CD19 targets. Wash steps are indicated by “[W]”. Data shows mean (± SD) % of live targets relative to NT T cells (**e**) or mean (± SD) IL-2 secretion (**f**) after 24 h. n = 3 donors from 1 experiment. Statistical analysis was through a one-way ANOVA with multiple comparisons between the 28BB-Fab-Tet-z CAR under different conditions. *P* values for cytotoxicity (**e**) were: 48 h wash versus no wash (*, 0.0118), 24 h wash versus no wash (**, 0.0050) and no drug versus no wash (**, 0.0044). *P* values for IL-2 secretion (**f**) were: 48 h wash versus no wash (**, 0.0015), no drug versus no wash (**, 0.0044), no drug versus 2 h wash (*, 0.0103) and 48 h wash versus 2 h wash (**, 0.0033).

The two most promising constructs (28-Fab-Tet-z and 28BB-Fab-Tet-z) were taken forward for more detailed characterization. To evaluate CAR inhibition over a range of minocycline concentrations, CAR T cells were cocultured at a 1:4 E:T ratio with SupT1-CD19-GFP with fourfold increasing doses of minocycline from 0.02 to 1600 nM. Here, cytotoxicity was compared relative to an inert TetCAR that could bind to CD19 but lacked any signaling capacity [constructs outlined in Supplementary Fig. S3(a) and (b)]. The inhibition of cytotoxicity to SupT1-CD19-GFP [Fig. 4a] increased with minocycline concentration up to 100 nM, at which point a plateau was reached. 28BB-Fab-Tet-z was more potently inhibited by minocycline, reaching 99% (± 31% SD) of live targets relative to the inert TetCAR, compared to 68% (± 44% SD) with 28-Tet-z. The IC_50_ was 4.5 nM for 28-Tet-z and 2.3 nM for 28BB-Tet-z. Both Fab-TetCARs tested also showed a similar dose-dependent reduction in both IFN-γ and IL-2 with increasing concentrations of minocycline, fully inhibiting cytokine secretion at concentrations > 6.25 nM [Fig. 4b,c]. The IC_50_ for 28-Fab-Tet-z and 28BB-Fab-Tet-z were 0.21 nM and 0.24 nM for IFN-γ and 0.34 nM and 0.44 nM for IL-2 secretion. In addition to IFN-γ and IL-2, secretion of a number of other effector cytokines by 28BB-Fab-Tet-z showed a similar dose-responsive decrease in response to minocycline [Supplementary Fig. S3(c)]. Lastly, inhibition of cytokine secretion by 28BB-Fab-Tet-z was also tested after addition of tetracycline and tigecycline, a glycylcycline derivative of tetracycline. Both small molecules inhibited IFN-γ and IL-2 secretion, however this required higher concentrations than minocycline (> 100 nM) and had no effect at lower doses [Supplementary Fig. S3(d) and (e)].

### Minocycline induces rapid and reversible inhibition of TetCAR signaling

To further examine the kinetics of minocycline-induced inhibition of TetCAR effector function, IL-2 secretion was assessed 1–5 h after co-culture with SupT1-CD19 targets. Each hour, 100 nM of minocycline was added to relevant wells [Fig. 4d]. As expected, the control CAR was unaffected by minocycline addition and induced detectable IL-2 secretion after 3 h. This was mirrored by 28BB-Fab-Tet-z in the absence of minocycline, however addition of minocycline at different time points was able to inhibit further cytokine secretion within 2–3 h. To ensure that the inhibition of TetCAR was reversible and that effector function could be restored upon removal of minocycline, 28BB-Fab-Tet-z cells were incubated overnight with 100 nM minocycline, then washed with media 48, 24 or 2 h before activation with SupT1-CD19 targets. Removal of minocycline 48 h before activation restored full TetCAR activity relative to a non-inhibited control, as measured by cytotoxicity and IL-2 secretion [Fig. 4e,f]. Washing at shorter timepoints (24 or 2 h) before activation only partially restored effector function.

### 28-Fab-Tet-z and 28BB-Fab-Tet-z CAR T cells are comparable with a gold-standard monolithic CAR

**Figure 5:**
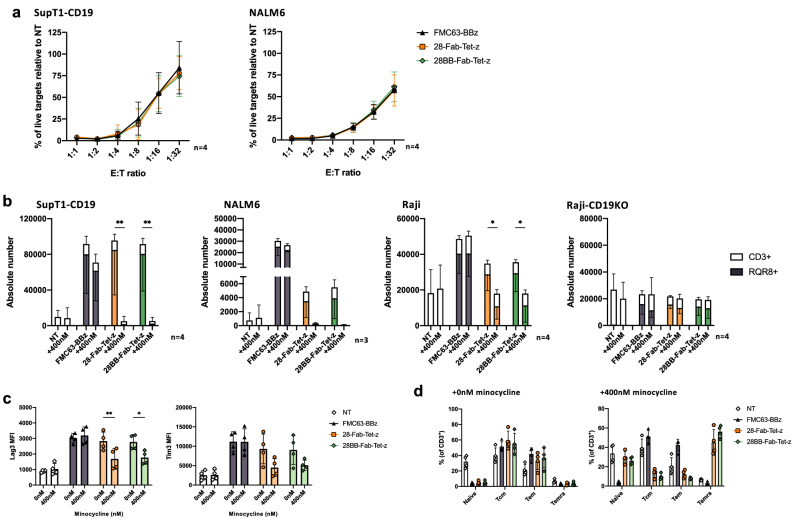
Effector function of CD28-containing TetCAR matches 41BBζ control CAR. (**a**) Killing of SupT1-CD19-GFP after 24 h or NALM6 after 48 h of co-culture with CAR-T cells at 1:1–1:32 E:T ratio. Data shows mean ± SD, n = 4 donors from 2 independent experiments. (**b**) SupT1-CD19, NALM6, Raji or Raji-CD19KO targets were incubated with mitomycin C, then co-cultured with CAR-T cells at 1:2 E:T ratio for 7 days. To relevant wells, 400 nM of minocycline was added on day 0. Graphs show mean (± SD) number of RQR8^+^ T cells (filled bars) or total CD3^+^ T cells (white bars) for each target. n = 4 donors (SupT1-CD19, Raji and Raji-CD19KO) or n = 3 (NALM6) from 2 independent experiments. Statistical analysis was through a two-way ANOVA comparing mean RQR8 number in each group ± minocycline (with Šidák’s multiple comparisons). *P* values for SupT1-CD19 were 28-Fab-Tet-z (**, 0.0028) and 28BB-Fab-Tet-z (**, 0.0050). *P* values for Raji were 28-Fab-Tet-z (*, 0.0320) and 28BB-Fab-Tet-z (*, 0.0304). (**c**) Mean fluorescent intensity of Tim3 and Lag3 after 7 days coculture with SupT1-CD19 targets, ± 400 nM minocycline. Data shows geometric mean (± SD) in CD3^+^ T cells. n = 4 donors, from 2 independent experiments. Statistical analysis was through a two-way ANOVA comparing each group ± minocycline (with Šidák’s multiple comparisons). *P* values for Lag3 expression were 28-Fab-Tet-z (**, 0.0078) and 28BB-Fab-Tet-z (*, 0.0207). (**d**) Percentage of naïve (CD62L^+^, CD45RA^+^), Tcm (central memory; CD62L^+^, CD45RA^-^), Tem (effector memory; CD62L^-^, CD45RA^-^) or Temra (terminally differentiated effector memory; CD62L^-^, CD45RA^+^) memory T cell populations after 7 days coculture with SupT1-CD19 targets, ± 400 nM minocycline. Data shows mean (± SD) in CD3^+^ T cells. n = 4 donors, from 2 independent experiments.

To ensure that 28-Fab-Tet-z and 28BB-Fab-Tet-z CAR T cells had no deficit in cell killing, we evaluated cytotoxicity at decreasing effector to target ratios, ranging from 1:1 to 1:32, to “stress” cytolytic function. There was no difference in cytotoxicity to SupT1-CD19-GFP or NALM6 at any E:T ratios in comparison to control CAR [Fig. 5a]. CAR T cell proliferation was also determined by co-culture with mitomycin C-treated SupT1-CD19-eGFP, NALM6, Raji cells or Raji CD19 knock-out cells [Fig. 5b]. In the absence of minocycline, both TetCARs proliferated to SupT1-CD19 similarly as the control CAR. However, in line with our observations for cytokine secretion, proliferation of the TetCARs was lower in response to NALM6 targets. The proliferative response of the TetCARs to Raji cells also appeared slightly lower than the control CAR, however this decrease was not significant. As expected, in the absence of CD19 on the Raji-CD19KO targets, none of the CARs proliferated above the NT T-cells. A single dose of 400 nM minocycline on day 0 was sufficient to significantly reduce TetCAR proliferation in response to SupT1-CD19 and Raji cells. A similar trend was observed with NALM6, however this was not significant due to poor response seen in the absence of minocycline in this setting.

### CD28 co-stimulation does not increase exhaustion or terminal differentiation in Fab-TetCAR T cells

Although CD28-CD3ζ endodomains can drive potent CAR T cell activation, this can limit memory formation and skew populations towards short-lived effector cells^22^. To show that Tet-CARs containing a CD28 endodomain were functionally equivalent to control 41BB-CD3ζ monolithic CAR, expression of Lag3/Tim3 or CD62L/CD45RA was evaluated. After the 7-day co-culture with target cells [Fig. 5c,d and Supplementary Fig. S4(a) and (b)], both Lag3 and Tim3 expression on CD3^+^ T cells were similar in the TetCARs compared with the standard CAR. Likewise, the proportions of the memory populations were similar between the TetCARs and the control CAR. Taken together these data show that even during maximal activation, TetCAR constructs containing CD28 endodomains did not drive an enhanced expression of exhaustion markers or skew differentiation of activated T cells.

### 28BB-Tet-CAR can be functionally regulated by minocycline in vivo

**Figure 6:**
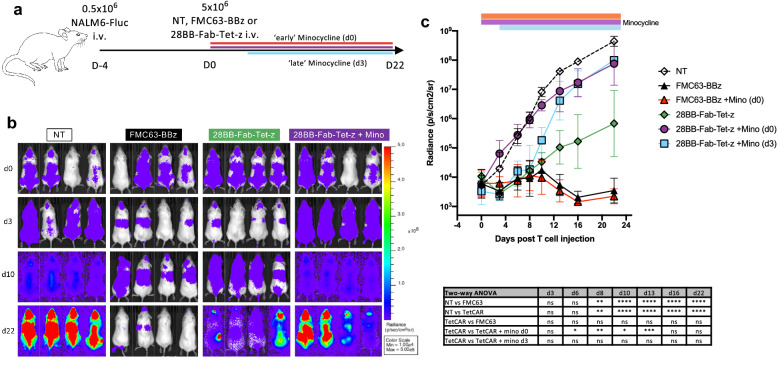
28BB-Fab-Tet-z function and inhibition in vivo. (**a**) Overview of in vivo experiment. NSG mice were injected i.v. with 0.5 × 10^6^ NALM6-FLuc tumor cells. On day 0, mice were randomly assigned based on tumor burden to receive 5 × 10^6^ non-transduced (NT), FMC63-BBz or 28BB-Fab-Tet-z CAR T cells. Groups were further divided with some to receive 0.4 mg minocycline i.p. every 1–2 days, starting either on day 0 or day 3. (**b**) Bioluminescence radiance (photons/s/cm^2^/sr) of NALM6-FLuc tumors in mice in select groups. (**c**) Geometric mean radiance (photons/s/cm^2^/sr) of NALM6-FLuc cells, in mice in all groups treated with NT, FMC63-BBz (± minocycline) or 28BB-Fab-Tet-z CAR T cells (± minocycline). n = 4 mice per group from 1 experiment. Table shows statistical analysis through one-way ANOVA with multiple comparisons between groups at each time point.

Lastly, we evaluated the activation and inhibition of 28BB-Fab-Tet-z CARs in vivo*.* NSG mice were engrafted with NALM6 engineered to express firefly luciferase (NALM6-Fluc); 4 days later, different cohorts were treated with either 5 × 10^6^ NT, FMC63-BBz, or 28BB-Fab-Tet-z CAR T cells, with or without minocycline. An additional cohort of 28BB-Fab-Tet-z treated mice were treated with minocycline 3 days after T cell transfer, during the peak of the initial anti-tumor response [Fig. 6a]. Minocycline was given at a dose of ~ 16 mg/kg (0.4 mg per mouse) i.p. every 1–2 days. There was a significant reduction in tumor burden with 28BB-Fab-Tet-z and FMC63-BBz versus NT T cells in the absence of minocycline [Fig. 6b,c]. However, there was a non-significant trend to shorter tumor control with 28BB-Fab-Tet-z, which resembled the reduced activation and proliferation of the TetCAR in response to NALM6 targets in vitro*.* Whilst the addition of minocycline on day 0 had no effect on the FMC63-BBz group, early inhibition of 28BB-Fab-Tet-z completely abrogated the tumor control seen in the absence of minocycline. Injection of minocycline after initial tumor control (on day 3) was also able to inhibit subsequent TetCAR activity. Overall, these data show that although 28BB-Fab-Tet-z CARs are less potent than the FMC63-BBz CAR, they nevertheless provide significant tumor control, which can be regulated by treatment with minocycline in a relevant tumor model in vivo*.* Furthermore, inhibition of TetCAR function can be initiated during and after CAR T cell activation in vivo*,* mirroring a clinically relevant application of this technology.

## Discussion

“Remote control” of CAR T cells was originally achieved by co-expression of suicide genes such as inducible Caspase-9^10,12^ or RQR8^11^. More recently, several CAR formats were described where activity can be controlled through small molecules^14–16^. Initial inducible CAR designs were split CARs where a small molecule was required to link antigen recognition and signaling domains. A more convenient format then followed where addition of the small molecule results in dissociation of the two domains, thereby inhibiting CAR activation^17^. Alternatively, addition of pharmaceutically inducible degron domains to the CAR protein also allows for controllable degradation of the CAR^23–25^. However, the small molecule drugs used to regulate the CAR T cells in the approaches noted above are often a key limitation in their widespread adoption. These often use unapproved, novel pharmaceuticals or in the case of the off switch CAR described previously^17^, the small molecule also binds to and inhibits the anti-apoptotic protein BCLxL.

Minocycline would be an ideal small molecule for control. Minocycline is a semi-synthetic tetracycline derivative, that is FDA approved, has no physiological binding partner and has been used clinically for over 30 years. Oral dosing is well tolerated and leads to rapid adsorption, with peak serum concentrations after 2 h and a nearly complete bioavailability^26–28^. Minocycline also has the added benefit that, unlike other analogues such as tetracycline, it is a highly lipophilic molecule that can readily cross the blood brain barrier, leading to high levels in the CSF and CNS^29^. This has potential importance for its use as a CAR safety switch due to the frequency of CAR-associated neurotoxicity/ICANS^6^.

To create a minocycline inhibited split CAR, we exploited the known binding of minocycline to the tetracycline repressor protein B (TetRB) and the short peptide mimotope of tetracycline, TIP^18,19^. We constructed a split CAR where the antigen binding domain contained an intracellular TetRB domain, while the signaling domain was expressed as a separate protein with an amino-terminal TIP sequence. We hypothesized that these two proteins would assemble, but that the higher affinity between TetRB and minocycline compared to TIP would allow inducible disruption of this protein–protein interaction. Initial designs showed effective displacement of this endodomain by minocycline and functional inhibition of TetCAR transduced cells. However, full effector function, as measured by cytokine secretion, was significantly lower than a monolithic control.

We made several empirical changes to improve TetCAR function. One successful modification was re-formatting the TetCAR into a Fab format. This format may have improved CAR function by improving stability, as evidenced by increased surface CAR staining. A further successful modification was the incorporation of co-stimulatory domains in the antigen-recognition component. Constructs in this format with membrane-proximal CD28, were able to match the cytokine secretion and proliferation of our gold-standard monolithic CAR in response to high-CD19 density targets. Membrane-proximal co-stimulation is also a feature of other split CAR approaches^14,17,25^, and so may be a requirement across all systems.

We were able to show that addition of minocycline impaired cytotoxicity and decreased cytokine secretion and proliferation to background levels. Minocycline was able to displace the signaling domain rapidly, leading to a functional inhibition of IL-2 secretion in less than 3 h. This inhibition was both reversible and dose dependent and was able to inhibit CAR signaling in a clinically relevant range of doses. Lastly, in an acute lymphoblastic leukemia model, administration of human PBMCs transduced with TetCAR into NSG mice bearing CD19-expressing NALM6 tumors was able to significantly reduce, but not clear tumor burden. Tumor control could be inhibited by i.p. injection of minocycline, either before or during initial tumor control. The sub-optimal tumor control against NALM6 in vivo reflects that, despite the optimization steps, maximal TetCAR activity to lower CD19 surface density targets showed significantly reduced cytokine secretion and proliferation compared to the monolithic CAR. However, impaired functionality was also seen in other regulatable CAR designs that failed to fully restore full effector function in some settings^17,23,25^. It remains a challenge to the field to have systems that maintain maximal CAR function whilst also conferring effective, inducible regulation of CAR activity.

A disadvantage of TetCAR is that the binding component, TetRB, is of bacterial origin, which may well be immunogenic. Notably, TetRB (and TIP) are intracellular, so not likely to be targets of humoral immunity. Attempts have been made to reduce the immunogenicity of dominant epitopes in the TetR protein^30^, however these have led to emergence of immune responses to sub-dominant epitopes, suggesting T cell immunity to TetRB may be unavoidable. However, the increasing use of allogeneic approaches where HLA is either disrupted^31^ or where completely mismatched cells are given in the setting of profound immunosuppression^32^ or allo-aggressive approaches^33^, may make cellular rejection moot. Future work will also focus on resolving the immunogenicity of TetRB, whilst preserving the benefits of minocycline as a small molecule, through replacing TetRB with other non-immunogenic binders of minocycline.

In summary, we have generated an ‘off’ switch CAR in which the activity can be rapidly, robustly and reversibly tuned down by dose dependent displacement of the CD3ζ signaling domain. The key benefit of this system over other similar approaches is that it utilizes a simple, well-understood, FDA-approved antibiotic that has excellent pharmacodynamics and blood brain barrier penetration. This system induced effective killing, cytokine secretion and proliferation in response to high antigen-density targets, while maintaining potent inhibition at clinically relevant minocycline doses. As inhibition of CAR signaling was rapid and reversible, this would allow administration of minocycline to patients in the event of CAR mediated toxicity, which could then be removed upon resolution of toxicity to restore CAR function. The approach described here would be most useful for either CARs directed at novel targets, particularly those with low-level expression on healthy tissue, or for future allo-approaches in CD19 malignancies.

## Materials and methods

### Cell lines

HEK-293 T were cultured in Iscove's modified Dulbecco's medium (IMDM) supplemented with 10% FBS and 2 mM GlutaMAX. SupT1, NALM6 and Raji lines were cultured in complete RPMI (RPMI-1640) supplemented with 10% FBS and 2 mM GlutaMAX. HEK-293 T, Raji and NALM6 lines were obtained from the American Type Culture Collection. SupT1 cells were purchased from the European Collection of Authenticated Cell Cultures. SupT1 were transduced with an SFG vector to express human CD19 and eGFP. NALM6 were transduced with an SFG vector to express firefly luciferase and a lipid-anchored hemagglutinin tag (HA-GPI). Raji-CD19KO (a gift from G.W-K. Cheung, University College London) were CRISPR edited to knock out CD19, then transduced with an SFG vector to express eGFP. Transduced cells were then single cell cloned by limiting dilution to establish a cell line.

### Retroviral transduction of T cells

RD114-pseudotyped γ-retroviral supernatants were generated by transfection of HEK-293 T cells with an SFG vector plasmid, an RD114 envelope expression plasmid (RDF, a gift from M. Collins, University College London), and a Gag-pol expression plasmid (PeqPam-env, a gift from E. Vanin, Baylor College of Medicine). Transfection was facilitated using GeneJuice. T cells from healthy donors were isolated by Ficoll gradient centrifugation and depleted of NK cells using CD56 microbeads (Miltenyi Biotec) according to manufacturer instructions. Cells were stimulated with 25 μl/ml ImmunoCult Human CD3/CD28 T Cell Activator (STEMCELL Technologies). IL-2 (GenScript) was added following overnight stimulation. On day 3, T cells were collected, plated on retronectin (Takara) with retroviral supernatant, and centrifuged at 1,000 g for 40 min. PBMCs were maintained in complete RPMI + 100 IU IL-2. Transduction efficiency was determined on day 5 after transduction, and further experiments were commenced on day 5–7 after transduction.

### Imaging of HEK-293 T expressing eGFP-CAR

Fluorescent microscopy was carried out in HEK-293 T cells transduced with the retroviral constructs. Prior to cell preparation, cover slips were treated with poly-L-lysine by incubating them for 1 h at room temperature and then drying them at 37 °C. The cover slips were washed with water and HBSS overnight at 4 °C. The next day, the cells were harvested and washed in HBSS and plated at 0.8 × 10^6^ per well in a 6-well plate, where they were incubated for 30 min at RT. The cell fluorescence was imaged with an Olympus BX63 fluorescent microscope at a × 40 lens magnification, and images were captured with an Olympus DP73 camera.

### Flow cytometry

Flow cytometry was performed using a cytoFLEX flow cytometer (Beckman Coulter). Staining steps were performed at 4 °C for 20 min, with PBS washes between steps. Cells were co-stained with eFluor 780 fixable viability dye (eBioscience). The following antibodies were used (anti-human; clone IDs are given in parentheses): CD2 (RPA-2.10), CD3 (UCHT1), Tim3 (F38-2E2), Lag3 (7H2-C65), CD45RA (HI100), CD4 (RPA-T4) and CD8 (RPA-T8) from BioLegend and CD62L (DREG-56) from BD Biosciences. CAR expression was detected by staining for the RQR8 marker with an anti-CD34 (Qbend10, R&D Systems) or by staining with a primary layer of soluble CD19 protein attached to a rabbit-Fc domain, followed by a goat anti-rabbit IgG antibody (Jackson Immunoresearch). Analysis was conducted using FlowJo v10 (Treestar).

### FACS-based killing assays

CAR transduced T cells were normalized based on RQR8-expression by dilution with non-transduced T cells. Target and effector cells were resuspended in complete RPMI and 50-100ul were added to U-bottom 96 well plates to achieve desired effector:target ratio. Different effector:target ratios were achieved through serial 1/2 dilutions of effector cells. Target cell number was kept constant within each experiment, ranging from 2.5–5.0 × 10^5^ cells. After 24–48 h, the plate was centrifuged at 400 g for 5 min, and 100 μl was removed for cytokine analysis. Cells were washed with 100 μl PBS and stained with viability dye. SupT1-CD19-GFP were distinguished from T cells by eGFP expression, whereas anti-CD2 was added to distinguish T cells from NALM6. CountBright beads (Thermo Fisher) were added to allow quantification of cells acquired. Gating on single live target cells was performed according to exclusion of fixable viability dye, forward and side scatter characteristics, and expression of eGFP or CD2. Assays were performed in duplicate. Percentage of live cells was calculated relative to the number of live target cells after co-culture with non-transduced T cells or inert-TetCAR T cells.

### Cytokine-release assays

Supernatants were removed from 24–48 h co-cultures of T cells and targets. IFN-γ and IL-2 concentration was determined by ELISA (BioLegend) according to manufacturer’s instructions. Further cytokine analysis was performed using the Essential Immune Response Panel Legendplex kit (BioLegend) according to manufacturer’s instructions.

### Inhibition with minocycline, tetracycline and tigecycline

Minocycline hydrochloride and Tetracycline hydrochloride (Sigma-Aldrich) were resuspended in water at 4 mg/ml, before dilution in RPMI to the required concentration. Tigecycline hydrate (Sigma-Aldrich) was resuspended in DMSO at 5 mg/ml, before dilution in RPMI. Stock solutions were made fresh for each experiment. Typically for 96-well plate assays, 50 μl of stock solution or cRPMI control was added per well.

### Reversibility assay

CAR-T cells were incubated overnight with 100 nM minocycline before being plated in 96-well plates. At 48, 24 and 2 h before addition of SupT1-CD19 targets, plates were spun and relevant wells were resuspended in fresh media. SupT1-CD19 targets were then added at a 1:1 E:T ratio. CAR effector function was assessed after 24 h through FACS based cytotoxicity and cytokine release assays as described above.

### Proliferation and exhaustion assays

Proliferation of CAR T cells was assessed by culturing T cells with target cells at a 1:2 ratio in duplicate in 96-well U-bottom plates. Targets were pre-treated with 4 μg/ml mitomycin C for 3 h, then washed 3 times with 30 ml RPMI before plating. After 7 days, cells were centrifuged at 400 g for 5 min, washed in PBS and stained with antibody cocktails to quantify number of RQR8^+^ CAR T cells. Antibodies to stain for exhaustion markers (Tim3 and Lag3) or differentiation markers (CD62L and CD45RA) were also added at this time.

### In vivo xenograft model with NALM6-FLuc

All animal studies were performed in accordance with a UK Home Office–approved project license and were approved by the University College London Biological Services Ethical Review Committee. Data from animal experiments are presented here in accordance with ARRRIVE guidelines. NSG mice (female, aged 6–10 weeks) were obtained from Charles River Laboratories and raised under pathogen-free conditions. Mice were intravenously injected with 0.5 × 10^6^ FLuc^+^ NALM6 (CD19^+^ acute lymphoblastic leukemia) and disease engraftment was assessed by bioluminescent imaging using the IVIS spectrum system (PerkinElmer) following intra-peritoneal injection of 2 mg D-luciferin. Photon emission from NALM6-FLuc cells, expressed in photon per second per cm^2^ per steradian, was quantified using Living Image software (PerkinElmer). Cohorts were randomized, and recipients with similar tumor burdens were distributed evenly across the groups prior to CAR T cell or non-transduced T cell injection on day 4 after tumor injection. Experiment was not blinded however bioluminescent imaging gives an objective value for tumour growth in this model. For mice treated with minocycline, a stock of 4 mg/ml minocycline hydrochloride was reconstituted in water before dilution in PBS to 2 mg/ml final concentration. 200 μl (0.4 mg) was injected intra-peritoneally every day for 2 weeks, then every 2 days afterwards. Mice were weighed every 2 days and animals with > 10% weight loss or those displaying evidence of graft-versus-host disease or disease progression, including hunched posture, poor coat condition, reduced mobility, piloerection or hindlimb paralysis, were killed.

### Statistical analysis

Unless otherwise stated, data are expressed as mean ± standard deviation, and analyses were performed in GraphPad Prism version 9 (San Diego, CA). Statistical analyses of in vitro assays were performed by two-way ANOVA with Tukey or Šidák post-test for multiple comparisons. For analysis with unequal variance between groups, these groups were removed from the analysis to allow comparison between select groups. This has been clearly highlighted in the figure legends. Significance of findings are defined as follows: NS, not significant; *P < 0.05; **P < 0.01; ***P < 0.001; ****P < 0.0001.

## Supplementary Information

Supplementary Information.

## Data Availability

The datasets generated during and/or analysed during the current study are available from the corresponding author on reasonable request.
