# Supplementary material for: Tunable control of CAR T cell activity through tetracycline mediated disruption of protein–protein interaction
Source: Sci Rep. 2021 Nov 9;11:21902. doi: 10.1038/s41598-021-01418-9 (PMC8578617; doi:10.1038/s41598-021-01418-9)
Supplement: Supplementary file 1 — Supplementary Information. [file 41598_2021_1418_MOESM1_ESM.pdf]

Supplementary Figures

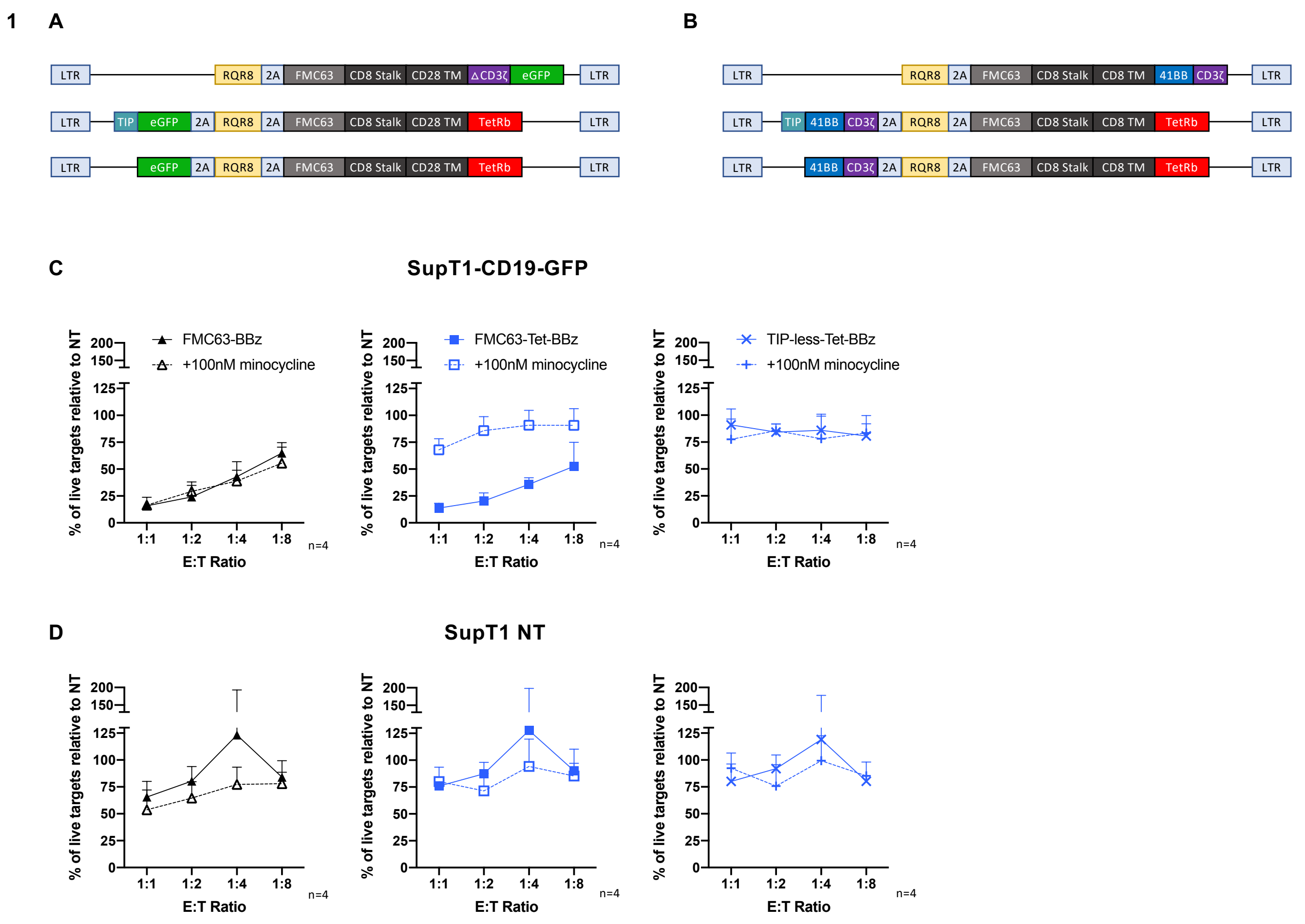

Supplementary Figure S1. Retroviral expression vectors and additional cytotoxicity assays

A) Schematic of retroviral vectors used to assess endodomain tagged-eGFP localization in different CAR architectures. All vectors contain the RQR8 sort/suicide gene, an anti-human CD19 scFv (FMC63, in a VL-VH orientation separated by a $(G_4S)_4$ serine glycine linker), human CD8 stalk region and human CD28 transmembrane domain. eGFP is either linked to the transmembrane domain after a truncated CD3ζ (first 5 amino acids) and short serine glycine linker (G_4S_2), or as a separate protein with or without a N-terminal TIP tag. TIP is linked to eGFP with G_3S_2 serine glycine linker. TetRB is linked to the CD28 transmembrane domain by a G_4S_2 serine glycine linker. T2A sequences were used to separate the different proteins.

B) Schematic of retroviral vectors encoding a standard 41BB-ζ CAR, or TetCAR with a split 41BB-ζ endodomain +/-TIP. All vectors contain the RQR8 sort/suicide gene, an anti-human CD19 scFv (FMC63), human CD8 stalk and transmembrane domains. 41BB-ζ domains are linked to the transmembrane domain or as a separate protein with or without the N-terminal TIP tag.

C,D) Killing of SupT1 cells engineered to express CD19 (C) or non-transduced (NT) (D) after 24 hours co-culture with CAR-T cells at a 1:1 - 1:8 effector:target ratio. 100nM of minocycline was added to relevant wells. Data shows mean percentage (±SD) of live cells compared to non-transduced (NT) T-cell control, n=4 donors from 1 experiment.

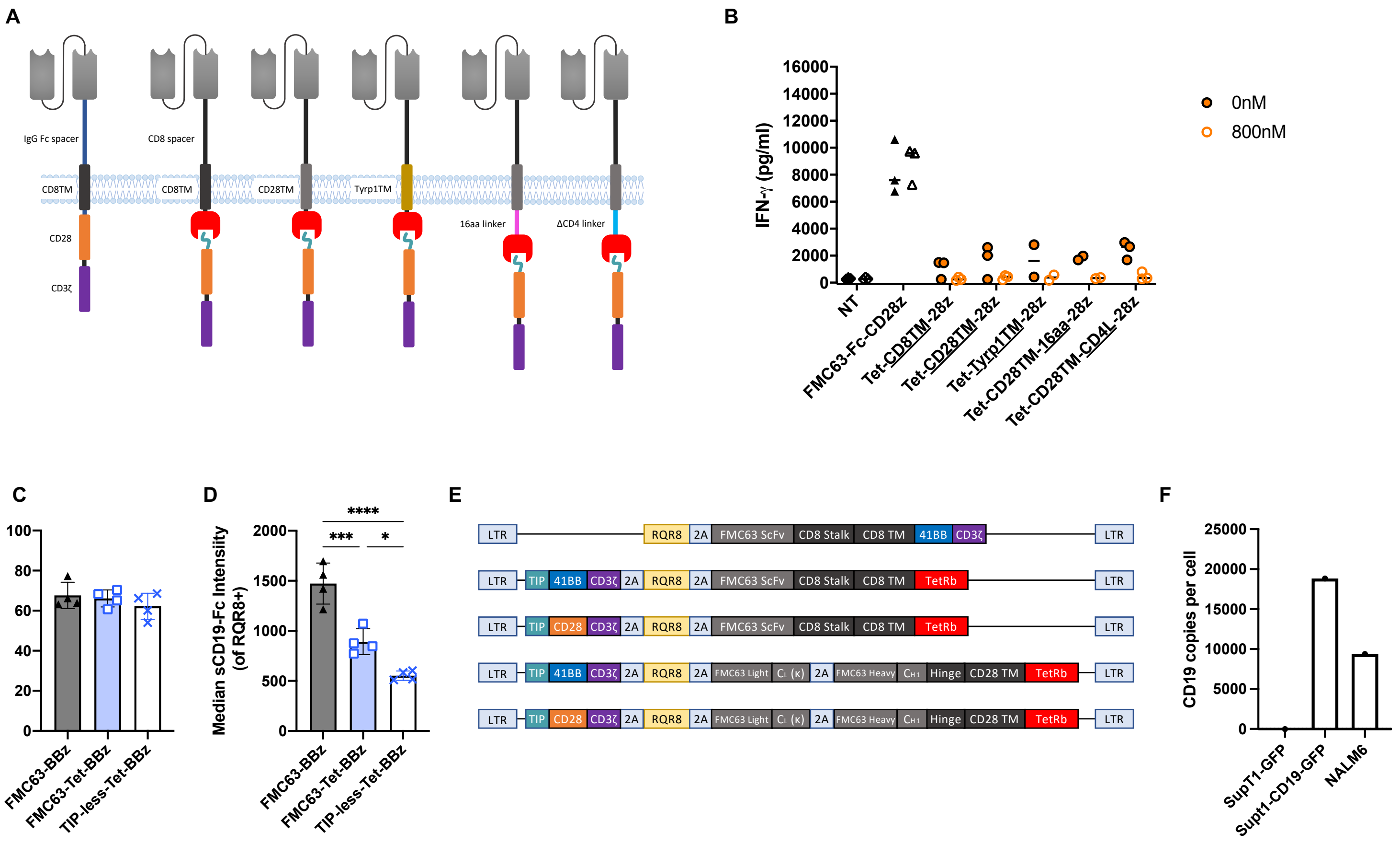

Supplementary Figure S2. Transmembrane domain and linker variants, retroviral expression vectors and CD19 expression on targets

A) Overview of the transmembrane (TM) and linker TetCAR variants with TIP-CD28-CD3ζ endodomains. The 16aa linker is a $(G_3S)_4$ serine glycine linker. The CD4 linker is the first 40 amino acids from the human CD4 intracellular domain.

B) IFN-γ release after 24 hours of co-culture with SKOV3 cells transduced with CD19 at 1:4 E:T ratio. 800nM minocycline was added to relevant wells. Data shows mean ±SD, n=2-3 donors from 1 experiment.

C) Transduction efficiency as measured by CD34 staining of the RQR8 marker gene. Bars show mean (±SD) % of RQR8$^+$ T cells, n=4 donors from 1 experiment.

D) Median fluorescent intensity of CAR expression on the surface of RQR8$^+$ cells as measured by staining with soluble, Fc-tagged CD19 protein. Bars show mean (±SD), n=4 donors from 1 experiment. Statistical analysis was through a one-way ANOVA with Tukey's multiple comparisons between each group. P-values were FMC63-BBz vs FMC63-Tet-BBz (***, 0.0007), FMC63-Tet-BBz vs TIP-less-Tet-BBz (*, 0.0205), and FMC63-BBz vs TIP-less-Tet-BBz (****, <0.0001).

E) Schematic of retroviral vectors encoding a standard 41BB-ζ CAR, or TetCARs with FMC63-scFv or Fab-derived binding domains with TIP-41BB-ζ or TIP-CD28-ζ endodomains. Fab-binding domains consist of the FMC63 variable light chain linked to the constant light kappa chain with a separate FMC63 variable heavy chain linked to the CH1 domain, which in turn is linked to the CD28 transmembrane domains via a hinge region.

F) Copies of CD19 per cell as measured after aCD19 staining and quantified using quantiBRITE beads.

3

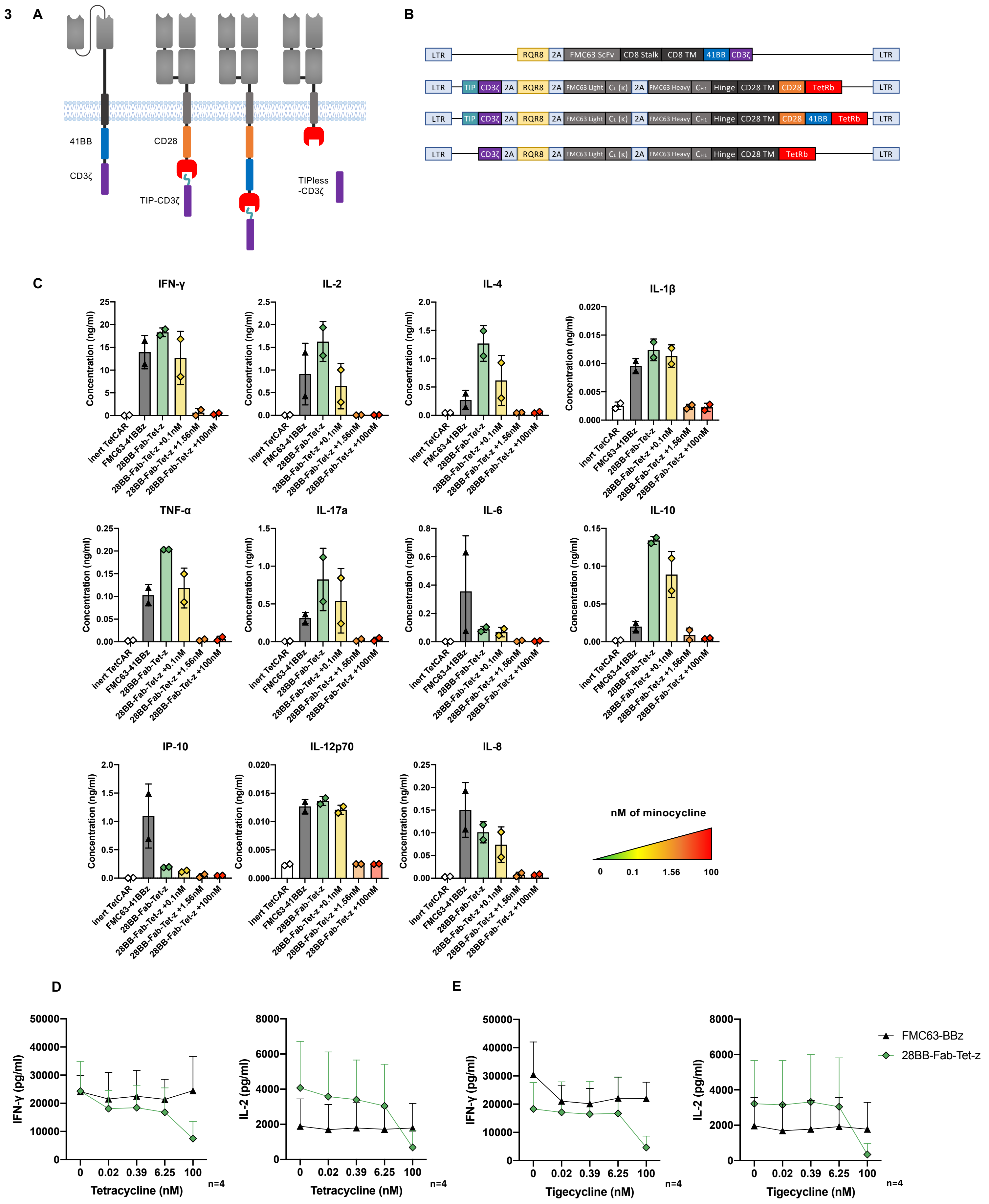

Supplementary Figure S3. 'Inert TetCAR' and retroviral expression vector overview, cytokine secretion panel and inhibition by tetracycline and tigecycline

A) Overview and schematic (B) of the standard CAR and Fab-TetCAR constructs used. An 'inert' TetCAR was constructed through removal of the costimulation domains (41BB and CD28) and inclusion of a 'TIP-less' CD3ζ domain.

C) Secretion of a range of cytokines by inert-TetCAR, FMC63-BBz or 28BB-Fab-Tet-z after 24 hours co-culture with SupT1-CD19. Inhibition of 28BB-Fab-Tet-z was assessed after addition of 0.1, 1.56 or 100nM minocycline. Data shows mean ±SD. 2 representative donors were picked based on IFN-γ and IL-2 release from figure 4(b) and (c).

D,E) IFN-γ and IL-2 release by FMC63-41BBz and 28BB-Fab-Tet-z after 24 hours of co-culture with SupT1-CD19 at various doses of tetracycline (D) or tigecycline (E). Data shows mean ±SD, n=4 donors from 1 experiment.

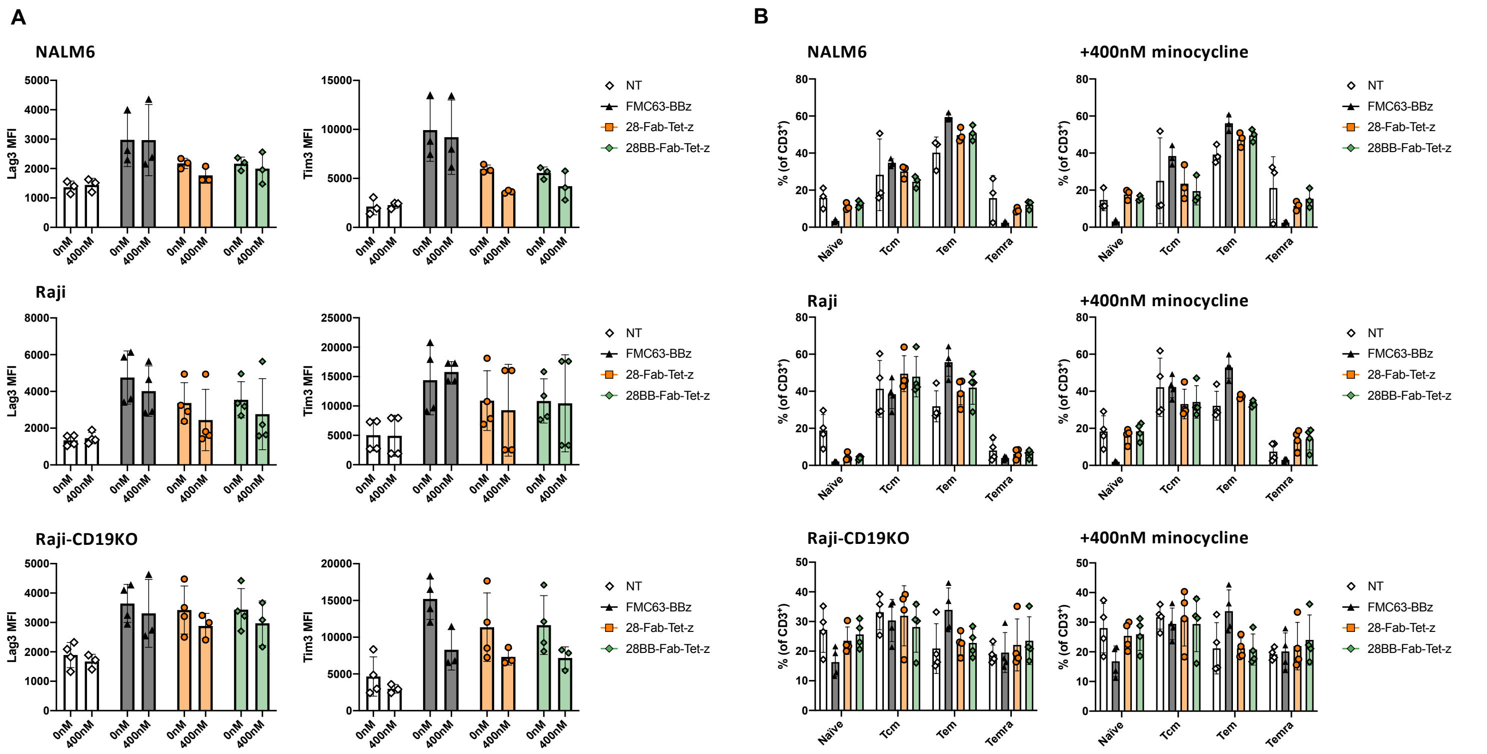

Supplementary Figure S4. Exhaustion and memory profile in response to NALM6 and Raji targets

A) NALM6, Raji or Raji-CD19KO targets were incubated with mitomycin C, then co-cultured with CAR-T cells at 1:2 E:T ratio for 7 days. To relevant wells, 400nM of minocycline was added on day 0. Data shows geometric mean fluorescent intensity of Tim3 and Lag3 (±SD) in $CD3^+$ T cells. n = 3-4 donors, from 2 independent experiments.

B) Percentage of naïve (CD62L+, CD45RA+), Tcm (CD62L+, CD45RA-), Tem (CD62L-, CD45RA-) or Temra (CD62L-, CD45RA+) memory T cell populations, after 7 days coculture with NALM6, Raji or Raji-CD19KO, ±400nM minocycline. Data shows mean (±SD) in CD3+ T cells. n = 3-4 donors, from 2 independent experiments.
